# Supplementary material for: Manuka Honey Reduces NETosis on an Electrospun Template Within a Therapeutic Window
Source: Polymers (Basel). 2020 Jun 26;12(6):1430. doi: 10.3390/polym12061430 (PMC7362002; doi:10.3390/polym12061430)
Supplement: Supplementary file 1 [file polymers-12-01430-s001.zip › Supplementary files 6 11 2020/Supplementary Document 1 Matlab code for Figures 5 and 6.docx]

% Load the images to be processed. Make sure they are in the directory

% folder containing the MATLAB code. Additionally, set the directory

% location in line 7 to contain the path to the folder where the images

% and code are saved. Change *.file to *.jpg for JPEGs or *.tif for TIFFs.

clc; % Clear the command window.

close all; % Close all figures (except those of imtool.)

clear; % Erase all existing variables. Or clearvars if you want.

workspace; % Make sure the workspace panel is showing.

image = dir('C:\Users\Ben\Documents\MATLAB\*.tif');

for i = 1 : length(image)

x = imread(image(i).name);

figure

imshow(x)

% Create the red pixel matrix and sets threshold. Makes another matrix

% for NET area with values below condensed nuclei but above another

% threshold

red=x(:,:,1);

rnuclei=red>=7000;

rnucs=red>=1000

rNETs=red>=7300;

% Create the green pixel matrix and sets threshold

green=x(:,:,2);

g=green>=5000;

% Create the blue pixel matrix.

blue=x(:,:,3);

b=blue>=7000;

% Create the red + blue (purple) pixel matrix (non-viable)

p=red>=3*blue;

nonv=rnuclei+p+b;

nonviab=nonv>=3;

% Create the red + blue + green (white) pixel matrix (NETosing)

w=rnucs+g+b;

NETosing=w>=3;

% Create the blue - green - red matrix (viable)

cyan=b+g;

c=cyan>=2;

viab=b-c-nonviab;

% Create the purple - green matrix (necrotic)

nec=nonviab-g;

necrotic_matrix=nec>=1;

% Create an image that shows the area identified as NETs

% Count number of NET pixels

NETs_matrix=rNETs-b;

warning('off', 'Images:initSize:adjustingMag');

NETs=mat2gray(NETs_matrix, [0 1]); C=imfuse(x,NETs); imshowpair(x,C,'montage');

num_NET_pixels=sum(NETs_matrix(:) == 1);

% Create an image that shows the area identified as rnuclei

%RNUCLEI=mat2gray(rnuclei); C=imfuse(x,RNUCLEI); imshowpair(x,C,'montage');

% Create an image that shows the area identified as green

%GREEN=mat2gray(g); C=imfuse(x,GREEN); imshowpair(x,C,'montage');

% Create an image that shows the area identified as blue

%BLUE=mat2gray(b); C=imfuse(x,b); imshowpair(x,C,'montage');

% Create an image that shows the area identified as nonviable nuclei

%NONVIAB=mat2gray(nonviab, [0 1]); C=imfuse(x,NONVIAB); imshowpair(x,C,'montage');

% Create an image that shows the area identified as NETosing nuclei

%NETNUCLEI=mat2gray(NETosing); C=imfuse(x,NETNUCLEI); imshowpair(x,C,'montage');

% Create an image that shows the area identified as viable nuclei

%VIAB=mat2gray(viab, [0 1]); C=imfuse(x,VIAB); imshowpair(x,C,'montage');

% Create an image that shows the area identified as necrotic nuclei

%NECROTIC=mat2gray(nec, [0 1]); C=imfuse(x,NECROTIC); imshowpair(x,C,'montage');

% Create an all black matrix and count the total number of

% analyzed pixels. The resulting variable "TotalPixels" should match the total

% number of pixels within your image.

AllBlack = zeros(size(x, 1), size(x, 2), 'uint8');

TotalPixels(i)=sum(AllBlack(:) == 0);

% Find percent area covered by NETs(images have 1920000 pixels)

PerAreaNETs(i)=num_NET_pixels/TotalPixels(i)*100;

%PERAREANETS=mat2gray(NETs_matrix, [0.1 0.9]); C=imfuse(x,PERAREANETS); imshowpair(x,C,'montage');

%for the viable pixel matrix containing DAPI but not SO/NE-stained pixels, concatenate

%the black (i.e. background) pixel matrix and viable pixel matrix. Convert

%to greyscale

Black_with_viab = cat(3, AllBlack, AllBlack, viab);

tenBlack_with_viab = Black_with_viab.*10;

ViabToGray=rgb2gray(tenBlack_with_viab);

% Binarize the greyscale image. Adjust luminance threshold as needed (threshold 0-1).

BinaryViab=imbinarize(ViabToGray,0.0);

% Select intact nuclei from binary image using size and circularity exclusion. Adjust

% upper and lower size limits as needed (preset values work for 20x image with an image

% 1600 x 1200 pixels). Display image of intact cell nuclei.

ViabNuclei=bwareafilt(BinaryViab,[40 800]);

[measuredImage,num_Viable_cells(i)]=bwlabel(ViabNuclei);

measurements=regionprops(measuredImage,'Area','Perimeter');

allAreas=[measurements.Area]

allPerimeters = [measurements.Perimeter]

circularities=allPerimeters.^2./(4*pi*allAreas)

keepers=circularities <1.3;

roundObjects=find(keepers);

binaryViabImage=ismember(measuredImage, roundObjects) > 0;

[measuredImage,num_Viable_cells(i)]=bwlabel(binaryViabImage);

%imshow(binaryViabImage);

% VIABLE=mat2gray(binaryViabImage, [0 0.9]); C=imfuse(x,VIABLE); imshowpair(x,C,'montage');

% For the NETosing pixel matrix containing NE + SO + DAPI-stained pixels, concatenate

% the black (i.e. background) pixel matrix and NETosing pixel matrix. Convert to

% grayscale.

Black_with_NETosing = cat(3, AllBlack, AllBlack, NETosing);

tenBlack_with_NETosing = Black_with_NETosing.*10;

NETosingToGray=rgb2gray(tenBlack_with_NETosing);

% Binarize the greyscale image. Adjust luminance threshold as needed (threshold 0-1).

BinaryNETosing=imbinarize(NETosingToGray,0);

% Select NETosing nuclei from binary image using size and circularity exclusion. Adjust

% upper and lower size limits as needed (preset values work for 20x image with an image

% 1600 x 1200 pixels). Display image of NETosing cell nuclei.

NETosingnuclei=bwareafilt(BinaryNETosing,[40 800]);

[measNNImage,num_NETosing_cells(i)]=bwlabel(NETosingnuclei);

allNNAreas=[measurements.Area]

allNNPerimeters = [measurements.Perimeter]

circularitiesNN=allNNPerimeters.^2./(4*pi*allNNAreas)

keepersNN=circularitiesNN <1.9;

roundObjectsNN=find(keepersNN);

binaryNNImage=ismember(measNNImage, roundObjectsNN) > 0;

[measNNImage,num_NETosing_cells(i)]=bwlabel(binaryNNImage);

%NETOSING=mat2gray(measNNImage, [0 1]); C=imfuse(x,NETOSING); imshowpair(x,C,'montage');

% For the necrotic pixel matrix containing DAPI and SO but not NE-stained pixels, concatenate

% the black (i.e. background) pixel matrix and green pixel matrix. Convert to

% grayscale.

Black_with_Nec=cat(3,AllBlack, AllBlack,nec);

bBlack_with_Nec=Black_with_Nec(:,:,3);

tenbBlac_with_Nec=Black_with_Nec.*10;

NecToGray=rgb2gray(tenbBlac_with_Nec);

%Binarize the greyscale image. Adjust luminance threshold as needed (threshold 0-1).

BinaryNec=imbinarize(NecToGray,0);

% Select the necrotic nuclei from binary image using size and circularity exclusion.

% Adjust upper and lower size limits as needed (preset values work for 20x image with an image

% 1600 x 1200 pixels). Display image of necrotic cell nuclei.

Necnuclei=bwareafilt(BinaryNec, [80 800]);

[measnecImage,num_Nec_cells(i)]=bwlabel(Necnuclei);

allnecAreas=[measurements.Area]

allnecPerimeters = [measurements.Perimeter]

circularitiesnec=allnecPerimeters.^2./(4*pi*allnecAreas)

keepersnec=circularitiesnec <1.5;

roundObjectsnec=find(keepersnec);

binarynecImage=ismember(measnecImage, roundObjectsnec) > 0;

[measNnecImage,num_Nec_cells(i)]=bwlabel(binarynecImage);

%NECROTIC=mat2gray(Necnuclei, [0 1]); C=imfuse(x,NECROTIC); imshowpair(x,C,'montage');

%Count number of Necrotic cell nuclei in the field of view.

[labeledImage,num_Nec_cells(i)]=bwlabel(Necnuclei);

end

% Create output data file named 'Data.' Save this variable to calculate the

% orange-to-green ratio.

ViableCells = num_Viable_cells';

NETosingCells = num_NETosing_cells';

NecroticCells = num_Nec_cells';

FinalPerAreaNETs = PerAreaNETs';

ImageNames=struct2table(image);

ImageNamesOnly=removevars(ImageNames,{'folder','date','bytes','isdir','datenum'});

DataComb = [ViableCells NETosingCells NecroticCells FinalPerAreaNETs];

colNames = {'ViableCells','NETosingCells','NecroticCells', 'FinalPerAreaNETs'};

DataTable = array2table(DataComb,'VariableNames',colNames);

%Data = [ImageNamesOnly DataTable];
