# Supplementary material for: Manuka Honey Reduces NETosis on an Electrospun Template Within a Therapeutic Window
Source: Polymers (Basel). 2020 Jun 26;12(6):1430. doi: 10.3390/polym12061430 (PMC7362002; doi:10.3390/polym12061430)
Supplement: Supplementary file 1 [file polymers-12-01430-s001.zip › Supplementary files 6 11 2020/Supplementary Figures.docx]

Figure S1. Zoomed-in views of the honey release from the 0.1% and 1% honey SD and LD samples. No significant differences were found at any timepoint between these samples.


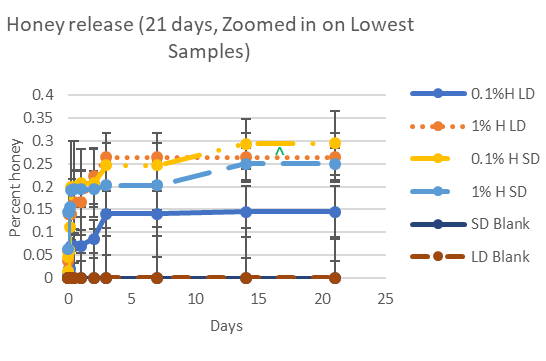

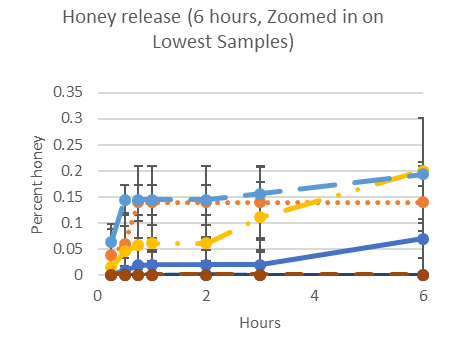


**Figure S2.** DAPI channel of images from Figure 4 in the text. Scale bars = 50 μm. Note: the nuclei are very small, so you may have to zoom in on the images to observe them.


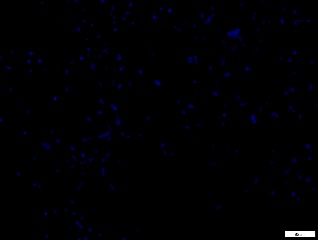

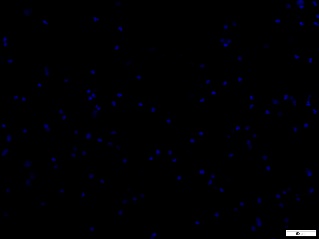

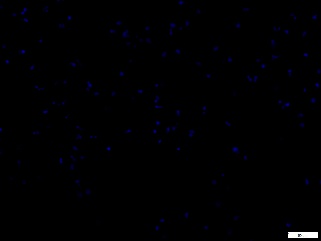

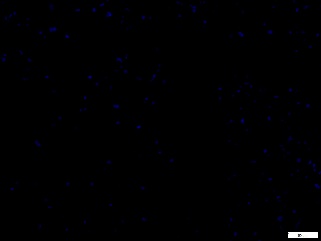

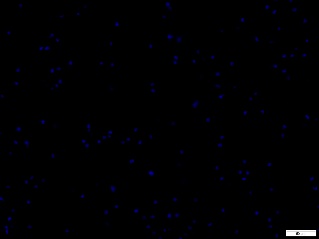

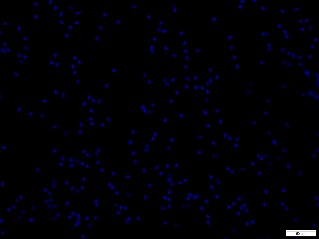

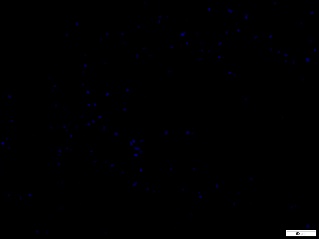

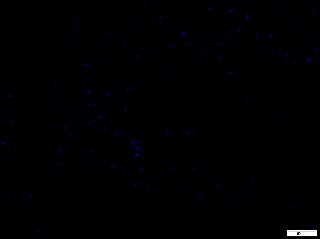

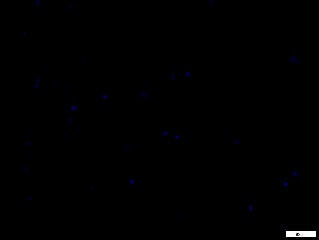

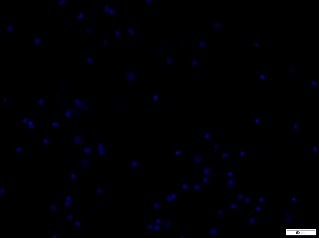

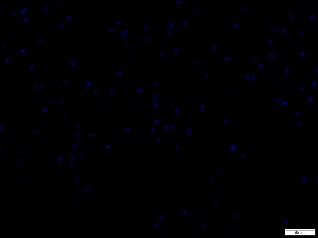

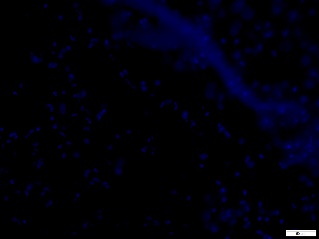

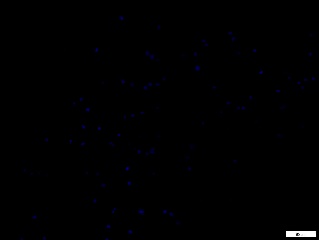

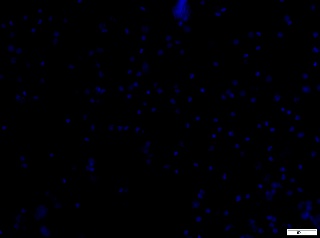

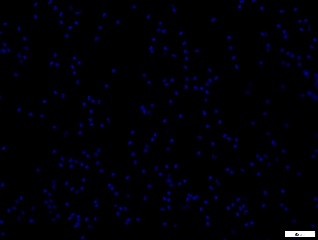

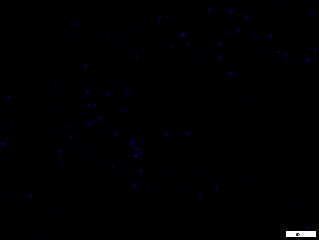


**10% Honey**

**1% Honey**

**0.1% Honey**

**Blank**

**3 Hours**

**LD**

**LD**

**6 Hours**

**SD**

**SD**

**10% Honey**

**1% Honey**

**0.1% Honey**

**Blank**

**3 Hours**

**LD**

**LD**

**6 Hours**

**SD**

**SD**


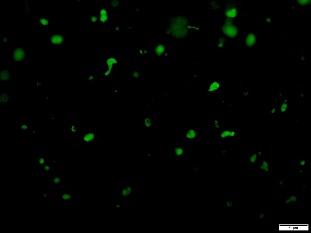

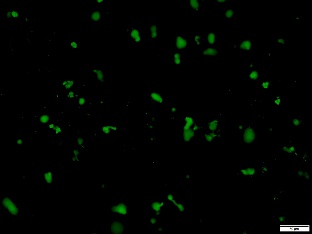

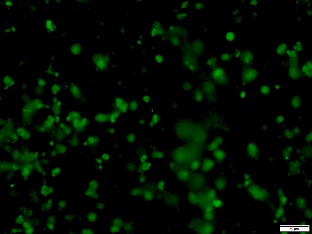

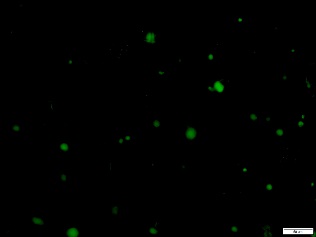

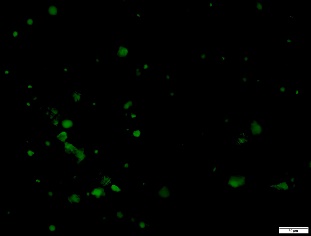

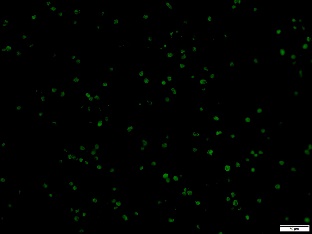

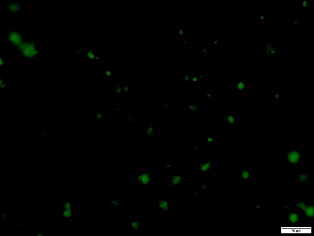

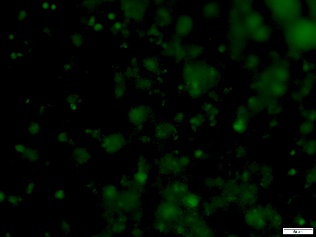

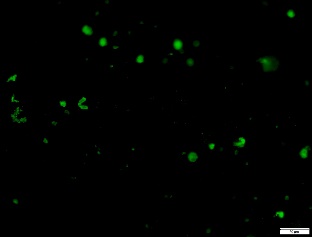

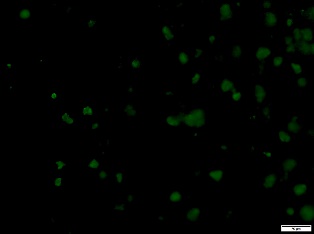

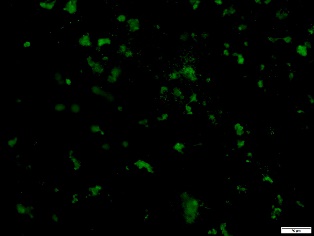

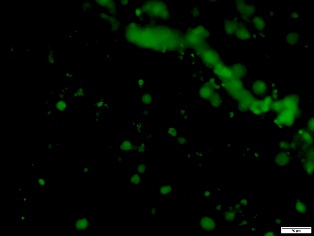

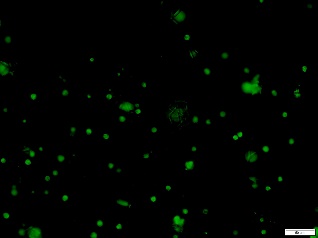

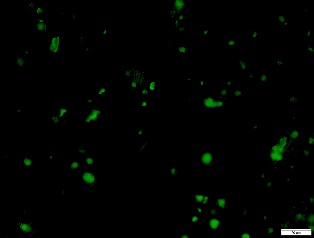

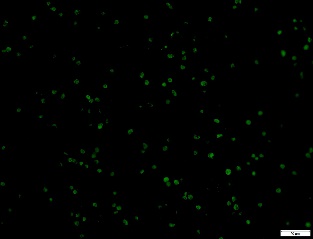

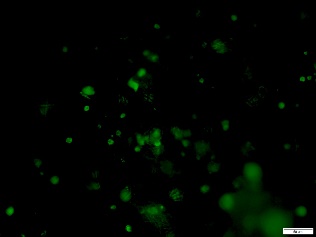


**Figure S3.** FITC channel of images from Figure 4 in the text (measuring Neutrophil Elastase immunostaining). Scale bars = 50 μm.

**Figure S4.** TRITC channel of images from Figure 4 in the text (measuring sytox orange staining of extracellular neutrophil DNA). Scale bars = 50 μm.


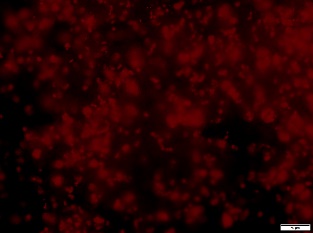

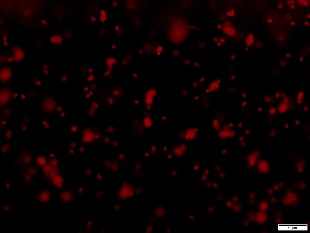

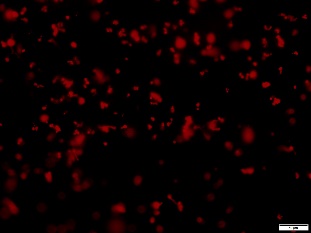

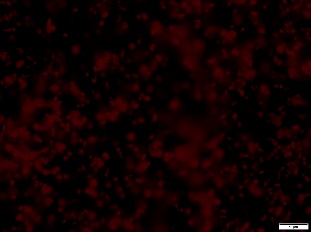

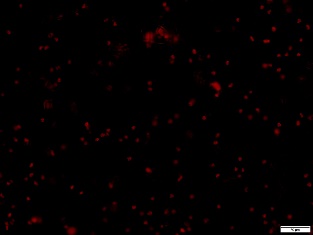

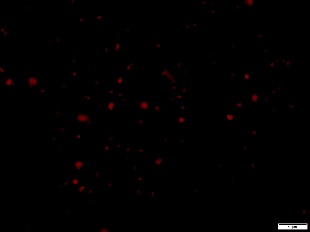

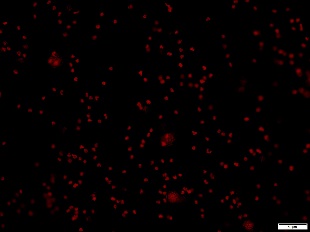

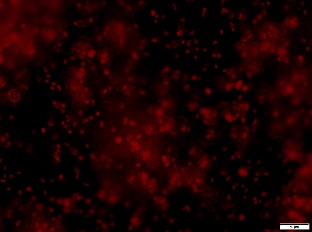

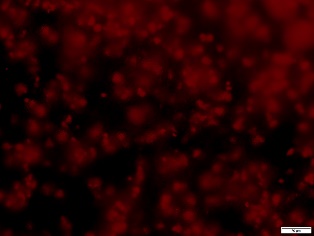

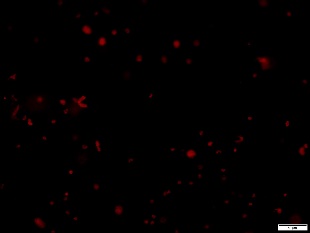

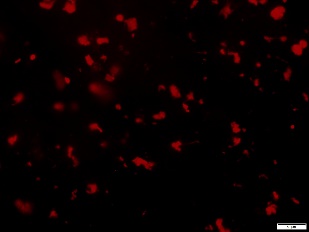

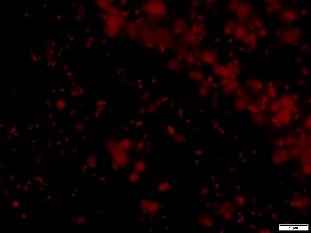

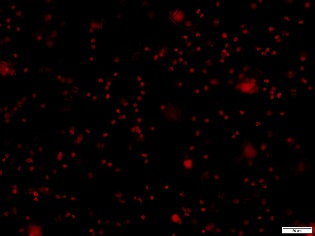

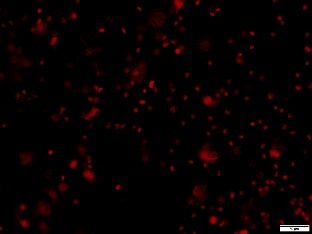

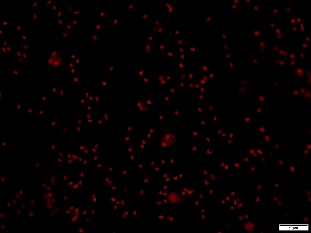

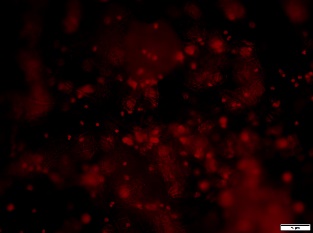


**10% Honey**

**1% Honey**

**0.1% Honey**

**Blank**

**3 Hours**

**LD**

**LD**

**6 Hours**

**SD**

**SD**
